# A Case of Tubercles of the Brain

**Published:** 1846-12

**Authors:** Wm. C. Sneed

**Affiliations:** Frankfort, Ky.


Art. III.—*A Case of Tubercles of the Brain.* By Wm. C. Sneed, M.D., of Frankfort, Ky.

The subject of this case was an inmate of the Kentucky penitentiary. He entered that institution about three years since, at which time he appeared to be a man of about thirty five years of age, and of good constitution; but he had been in the habit of indulging freely in ardent spirits. About two years since he entered the hospital of the prison, with some disease which we could not at that time clearly distinguish; he was feeble, complained of headache, loss of appetite, and general debility. After remaining in the hospital three or four weeks, and occasionally taking a little medicine, he recovered sufficiently to resume his occupation (that of *filling*

*spinner*), and so continued up to his last illness. About five weeks since he entered the hospital with diarrhœa and was placed under treatment for that disease; in a few days, however, he was relieved, and was placed by the keeper as a laborer in the stone-quarry near the walls of the prison, where he was exposed to the sun during the intensely hot weather in the early part of July.

In a few days he came again to the hospital complaining of great general debility and a pain in the front part of his head. Suspecting typhoid fever, we had his bowels evacuated, placed him upon rice water as a diet, and administered some mild diaphoretic medicine. He continued to emaciate, lost appetite, and remained in a half lethargic state up to the time of his death.

The pulse was slow and regular during the whole of the attack, varying from 55 to 70 beats in a miuute; the tongue generally coated white, but never dry; there were no ner vous symptoms, no paralysis, no delirium, nor symptom of any kind that indicated active disease of the brain. During the early stage of the attack the pupils of the eyes were somewhat contracted, but shortly before death slightly dilated; there was no indication of fever, nor any stupor; but he was able to distinguish and answer questions propounded the day before his death, which occurred on the 13th inst.

*Autopsy six hours after death.*—The abdomen was first opened, and the bowels found in a very contracted condition but otherwise healthy; the mesenteric glands were slightly enlarged and indurated, and the assisting chylopoietic viscera apparently healthy.

The thorax was next opened, and both lungs found adherent to the parietes of the chest, the left more extensively than the right, and both filled with crude undeveloped tubercles, very generally dispersed and more numerous in the right lung. Nothing being found in either of these cavities sufficient to produce death, we were led to the brain, confidently expecting that in that viscus some lesion would present itself sufficient to produce it.

On removing the above, the glandulæ pacchaoni were found numerous and large, but none softened or suppurated; a collection of pus occupied a space the size of an eagle half dollar, to the right of the median line, and near the upper posterior angle of the right parietal bone; the dura mater appeared sound when the pus was removed, but the ulcer had extended nearly through the inner table of the skull.

Whether this ulcer was produced by caries of the inner table primarily, or by the softening of one of the glands, we were unable to determine; but caries existed and presented a phenomenon rarely met with. The dura mater was removed and found adherent to the pia mater at the frontal sinus, but no other part; and the arachnoid studded with tubercles, small but hard. Externally, the brain appeared natural, but a section of it presented an immense number of tubercles; in the cortical substance they were large and hard, but in the cineritious many had softened, and their places were occupied by yellow pus. They were not grouped, but generally dispersed; they were usually the size of a pea, and occupied almost every portion of the organ—both hemispheres, the corpora striata, thalami optici, medulla oblongata, crura cerebri and cerebelli. The ventricles contained more fluid than natural, but no evidences of recent inflammation of the brain were found.

The history of the case, together with the appearances found in the post-mortem examination, would lead me to the following conclusions:

1st. The disease had been approaching for years, but so gradually and slowly as not to give rise to any active symptoms.

2d. That it was of a scrofulous character, and would ultimately have attacked either the lungs or mesenteric glands, and in that way produced death.

It is remarkable that the formation and softening of so many tubercles, did not give rise to more active symptoms than were present during his illness. We are at a loss to account for the slowness of the pulse, the absence of paralysis and fever, the rapid emaciation, and the clearness of his mind.

August 20th, 1846.

